# Association of type 2 diabetes mellitus with histopathological features of Non-metastatic breast cancer in Chinese women: a retrospective Cross-sectional study

**DOI:** 10.1038/s41598-025-14077-x

**Published:** 2025-08-05

**Authors:** Lei Liu, Ying Gao, Peng Liu, Rui Hui, Jin Zhang

**Affiliations:** 1 The 3rd Department of Breast Cancer, Tianjin Medical University Cancer Institute and Hospital, National Clinical Research Center for Cancer, Key Laboratory of Cancer Prevention and Therapy, Tianjin, 300060 China; 2 Tianjin’s Clinical Research Center for Cancer, Key Laboratory of Breast Cancer Prevention and Therapy, Tianjin Medical University, Ministry of Education, Tianjin, 300060 China; 3 Department of Breast and Thyroid Surgery, Tianjin Key Laboratory of General Surgery in Construction, Tianjin Union Medical Center, The First Affiliated Hospital of Nankai University, No. 190 Jieyuan Road, Hongqiao District, Tianjin, 300121 China

**Keywords:** Breast cancer, Molecular subtype, Type 2 diabetes mellitus, Glycemic control, Histopathology, Diseases, Health care, Medical research

## Abstract

Breast cancer and type 2 diabetes mellitus (T2DM) are prevalent global health concerns, often sharing overlapping pathophysiological mechanisms. This study aimed to characterize the association of type 2 diabetes mellitus (T2DM) with the histopathological features of non-metastatic breast cancer in Chinese women and evaluate whether glycemic control, diabetes duration, and treatment were involved. A retrospective cross-sectional analysis was conducted on 924 patients with stage I–III ductal breast cancer, equally divided into diabetic (*n* = 462) and nondiabetic (*n* = 462) groups. Patients were stratified by fasting blood glucose (FBG) levels, use of metformin and insulin, and diabetes duration. The expression of hormone receptors estrogen and progesterone (ER, PR), human epidermal growth factor receptor 2 (HER2), Ki-67, histological grade, lymph node metastasis, and pathological tumor node metastasis (pTNM) staging were analyzed. Diabetic patients exhibited a higher incidence of triple-negative breast cancer (TNBC) and a lower incidence of HER2-positive breast cancer compared to nondiabetic patients. Poor glycemic control (FBG ≥ 10 mmol/L) was associated with a higher risk of TNBC, histological grade III disease, and lower ER-positive rates compared to those with FBG < 6.1 mmol/L. Patients taking metformin had an increased likelihood of TNBC and decreased ER-positive rates. Insulin-treated patients demonstrated a lower prevalence of pTNM stage I cancers but a nearly fivefold increase in ductal carcinoma in situ (DCIS). Those receiving combination treatment of metformin and insulin were also more likely to present with DCIS. Patients with a longer diabetes duration (≥ 7 years) had significantly lower risks of TNBC and HER2-positive breast cancer but were more likely to have ER- and PR-positive subtypes and histological grade I tumors. These findings highlight the impact of T2DM, glycemic control, and diabetes management on the molecular and histopathological features of non-metastatic breast cancer. While associations were identified, causality could not be determined due to the cross-sectional design. Understanding these associations could guide tailored treatment strategies for diabetic breast cancer patients.

## Introduction

Breast cancer is the most commonly diagnosed malignancy among women worldwide, accounting for approximately a quarter of all cancer cases in women^1^. It is also the leading cause of cancer-related mortality among women^2^. Type 2 diabetes mellitus (T2DM), similarly, is a growing public health concern, particularly among aging women, with overweight and obesity recognized as major contributing risk factors. In China, breast cancer is the most prevalent malignancy in women, with an incidence of 63.3 cases per 100,000^3^. The age-standardized incidence of T2DM in Chinese women is approximately 9.2 per 1000 person-years, as reported in a 2010 prospective study^4^.

Epidemiological evidence increasingly supports a link between diabetes and breast cancer, including a heightened risk of disease onset and a worsened prognosis^5–8^. Several biological mechanisms have been proposed to explain this association, including hyperinsulinemia, chronic inflammation, dysregulation of endogenous sex hormones, and activation of insulin and insulin-like growth factor (IGF) signaling pathways^9^. While the link between diabetes and breast cancer risk is well supported, its relationship with molecular subtypes and histopathological features of breast cancer remains incompletely characterized.

Antidiabetic medications may further influence breast cancer biology. Metformin, a first-line therapy for T2DM, improves insulin sensitivity and has demonstrated potential anti-tumor activity in experimental models by inhibiting the mammalian target of rapamycin (mTOR) signaling pathway^10–14^. In contrast, exogenous insulin may activate mitogenic pathways and promote tumor cell proliferation and angiogenesis via IGF-1 receptor activation^15^. Previous studies exploring the associations between T2DM, antidiabetic treatment, and breast cancer subtypes have yielded inconsistent results^16,17^. Moreover, most research has focused on long-term outcomes rather than the tumor’s molecular and pathological features at diagnosis. In this retrospective cross-sectional study, we aimed to examine the association between T2DM and histopathological features of breast cancer in Chinese women. We further assessed whether glycemic control, diabetes duration, and treatment (metformin and/or insulin) were related to specific molecular subtypes. In this study, early-stage breast cancer was defined as non-metastatic invasive ductal carcinoma corresponding to American Joint Committee on Cancer (AJCC) stages I through III. Given the observational nature of our design, we also recognize that any associations observed cannot be interpreted as causal and may be influenced by confounding factors such as age, obesity, and metabolic syndrome.

## Methods

### Patient selection and study design

**Fig. 1:**
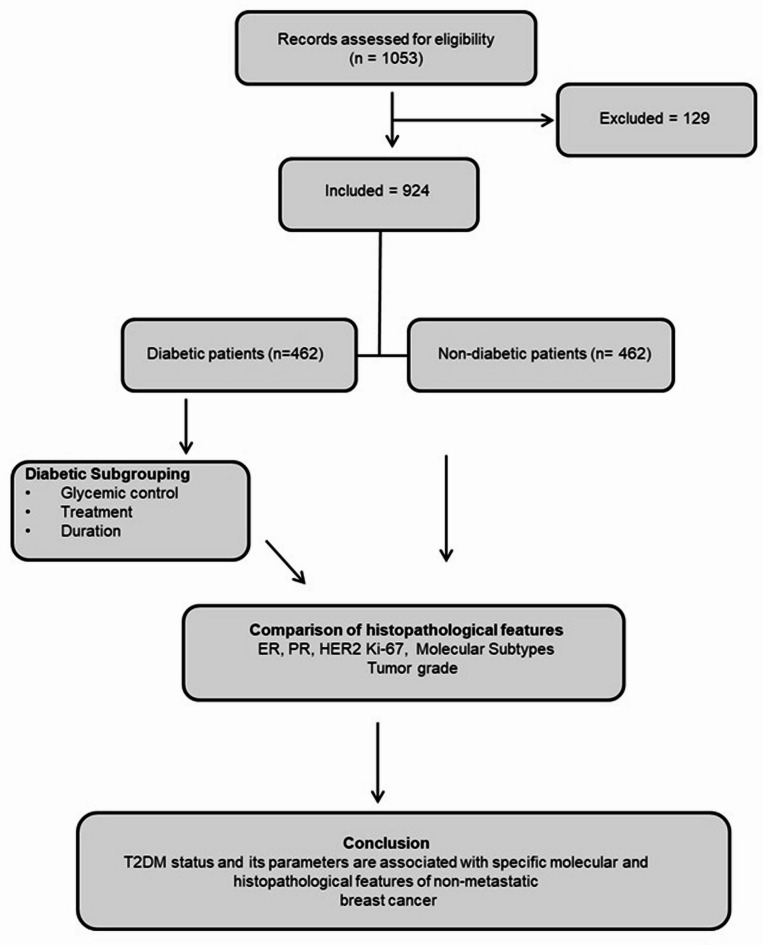
Study flow diagram illustrating patient selection, diabetic subgroup stratification, and analysis of histopathological features and molecular subtypes in relation to glycemic control, treatment, and diabetes duration.

Cases of stage I–III breast cancer which were surgically treated, without neoadjuvant chemotherapy, in the Breast Cancer Center of Tianjin Medical University Cancer Institute and Hospital between July 2013 and January 2018 were retrospectively studied. The sample size was determined by the availability of complete clinicopathologic records in the institutional database. Power calculations were not performed due to the retrospective cross-sectional design. Patients were selected based on their diabetic status and hypoglycemic treatments at the time of breast cancer diagnosis, as well as a range of histopathological characteristics, such as medical history, family history, medications, etc., which were collected at the time of the first visit and archived into a database. Previous medical records were also archived when available. The flow diagram of the study is presented in Fig. 1.

An equal number of non-diabetic patients were selected using stratified random sampling from the same database, matched by diagnosis period and completeness of clinical records, and falling outside the exclusion criteria. Patients with lobular or any other specific type of carcinoma, stage IV disease at diagnosis, stage I–III disease but treated by neoadjuvant chemotherapy before surgery, or bilateral breast cancer were excluded. Patients with pregnancy or lactation were also excluded. Patients with type 1 diabetes mellitus or without complete and accurate information on diabetic treatment or history were excluded from the diabetic group. Patients with fasting blood glucose (FBG) levels over 6.1 mmol/L at diagnosis were excluded from the non-diabetic group. This study was approved by the ethical committee of Tianjin Medical University Cancer Institute and Hospital under approval number TMC/H/09/18. The research adheres to the Declaration of Helsinki and that all research was performed in accordance with relevant guidelines/regulations. Due to the retrospective nature of the study, ethical committee of Tianjin Medical University Cancer Institute and Hospital waived the need of obtaining informed consent.

### Tumor analyses

In this study, the pathological reports of the routine surgical specimens were used. Tumor, node, and metastasis staging of breast cancer was performed following the AJCC Cancer Staging Manual (8th edition, 2016), and the histological classification was assigned based on the World Health Organization classification system. Tumors were graded using the modified Black’s nuclear grading system.

Molecular classification was based on the expression of estrogen receptor (ER), progesterone receptor (PR), Ki-67, and human epidermal growth factor receptor 2 (HER2), following the definition of individual subtypes recommended by the St. Gallen Breast Cancer Consensus 2017^18^. Expression of ER, PR, and Ki-67 was examined by immunohistochemistry and scored by pathologists. Sections with ≥ 1% of cells expressing ER or PR were scored as positive. Ki-67 staining was scored as the percentage of cells with positive nuclear signals (0–100%), and sections with > 20% of positive cells were defined as having a high level of Ki-67 expression. HER2 positivity was examined by immunohistochemistry and fluorescence in situ hybridization (FISH). The tumors were considered HER2-positive if the immunohistochemical score was 3 + or HER2 gene amplification was confirmed by FISH, according to the American Society of Clinical Oncology guidelines.

### Diabetes evaluation

To evaluate the association of diabetes with the incidence and molecular subtype of breast cancer, patients were subgrouped based on their glycemic control, which was assessed by measuring the fasting blood glucose (FBG) level at their first admission to the hospital. A FBG value < 6.1 mmol/L was considered good glycemic control, 6.1–9.9 mmol/L as moderate, and ≥ 10.0 mmol/L as poor glycemic control, based on thresholds used by the World Health Organization and the Chinese Diabetes Society^18^.

In addition, patients were classified based on whether metformin or insulin was used for the treatment of diabetes. Patients were also classified into a metformin-insulin combination subgroup and a subgroup with neither treatment. Furthermore, the association of breast cancer characteristics with diabetes duration was assessed. Duration of diabetes was defined as the interval from diagnosis of diabetes to diagnosis of breast cancer, and 7 years was used as the cutoff point, based on the median duration within our cohort and prior literature suggesting prognostic differences at this threshold^19^.

### Statistical analysis

Data were processed using SPSS version 22.0 (Chicago, IL, USA). Categorical variables, including menopausal status, ER, PR, HER2, Ki-67, molecular subtype, histological grade, lymph node metastasis, and pathological TNM staging, were compared between diabetic and non-diabetic groups using the Chi-square test. Differences in age between groups were analyzed using the Mann–Whitney U test due to non-normal distribution. Multivariate logistic regression was conducted to evaluate independent associations of glycemic control, diabetes duration, and treatment type (metformin and/or insulin) with breast cancer subtypes. The models were adjusted for age and menopausal status to reduce potential confounding. A p-value < 0.05 was considered statistically significant. Patients with missing data on key clinicopathological variables were excluded from the respective analyses. No imputation techniques were applied. Sensitivity analyses were not performed, as the retrospective study design and available dataset did not permit subgroup validation or alternative analytical modeling.

## Results

### Diabetes is associated with the incidences of HER2-positive breast cancer and TNBC

A total of 924 patients, including 462 diabetic and 462 nondiabetic patients with ductal breast cancer, were included in this retrospective cross-sectional study. The clinico-pathological characteristics of these patients are summarized in Table 1. The diabetic group had a significantly higher median age (60.9 vs. 52.8 years; *p* < 0.001) and a significantly higher percentage of postmenopausal women (84.8% vs. 58.4%; *p* < 0.001) compared to the nondiabetic group. Diabetic status was significantly associated with the molecular subtype of breast cancer. A higher incidence of triple-negative breast cancer (TNBC) was observed in the diabetic group compared to the nondiabetic group (20.3% vs. 15.2%; *p* = 0.039), while the HER2-positive rate was lower (8.7% vs. 12.8%; *p* = 0.043). Additionally, diabetic patients were less likely to present with pTNM stage I disease (25.1% vs. 35.7%; *p* < 0.001) and more likely to be diagnosed with pTNM stage IIa (39.2% vs. 28.1%; *p* < 0.001). However, there were no significant differences in the expression of ER, PR, Ki-67, histological grade, or lymph node metastasis between diabetic and nondiabetic patients (Table 1).

**Table 1:** Clinico-pathological characteristics of the included breast cancer patients.

Characteristics	Diabetes (*n* = 462)	No diabetes (*n* = 462)	*p*
Median age (years)	60.9 (42.0–74.0)	52.8 (32.0–73.0)	< 0.001^a^
*Menopausal status*			
Pre	70 (15.2%)	192 (41.6%)	< 0.001^a^
Post	392 (84.8%)	270 (58.4%)	
*ER*			
Positive	343 (74.2%)	351 (76.0%)	0.543
Negative	119 (25.8%)	111 (24.0%)	
*PR*			
Positive	304 (65.8%)	287 (62.1%)	0.244
Negative	158 (34.2%)	175 (37.9%)	
*HER2*			
Positive	40 (8.7%)	59 (12.8%)	0.043^a^
Negative	422 (91.3%)	403 (87.3%)	
*Ki-67*			
High	339 (73.4%)	353 (76.4%)	0.288
Low	123 (26.6%)	109 (23.6%)	
*Molecular subtype*			
Luminal A	89 (19.3%)	84 (18.2%)	0.673
Luminal B	251 (54.3%)	271 (58.7%)	0.184
HER2	28 (6.1%)	37 (8.0%)	0.247
TNBC	94 (20.3%)	70 (15.2%)	0.039^a^
*Histological grade*			
I	21 (4.5%)	15 (3.2%)	0.308
II	328 (73.2%)	355 (76.8%)	0.197
III	103 (22.3%)	92 (19.9%)	0.375
*Lymph node metastasis*			
Positive	191 (41.3%)	172 (37.2%)	0.201
Negative	271 (58.7%)	290 (62.8%)	
*Pathological TNM staging*			
0 (DCIS)	23 (5.0%)	16 (3.5%)	0.185
I	116 (25.1%)	165 (35.7%)	< 0.001^a^
IIa	181 (39.2%)	130 (28.1%)	< 0.001^a^
IIb	64 (13.9%)	54 (11.7%)	0.324
IIIa	35 (7.6%)	40 (8.7%)	0.547
IIIb	1 (0.2%)	0 (0%)	1.000
IIIc	42 (9.1%)	57 (12.3%)	0.111

Data presented as median (range) or n (%).

^a^Statistically significant difference.

### Poor glycemic control correlates with the incidence of TNBC and advanced histological grade

To further investigate this association, diabetic patients were subclassified based on their fasting blood glucose (FBG) levels. Among the 125 diabetic patients with good glycemic control (FBG < 6.1 mmol/L), the molecular and histopathological profiles were similar to those of nondiabetic patients. When compared to diabetic patients with moderate glycemic control (6.1 ≤ FBG < 10.0 mmol/L), the only notable difference was a lower frequency of histological grade III tumors (15.2% vs. 24.2%; *p* = 0.040). However, diabetic patients with poor glycemic control (FBG ≥ 10 mmol/L) exhibited nearly twice the incidence of TNBC (37.5% vs. 19.2%; *p* = 0.012) and a higher proportion of grade III tumors (29.2% vs. 15.2%; *p* = 0.036) compared to those with good glycemic control. These patients also had significantly lower ER positivity (56.2% vs. 74.4%; *p* = 0.020) and were less likely to present with pTNM stage I disease (14.6% vs. 29.6%; *p* = 0.042) (Table 2).

**Table 2:** Characteristics of diabetic breast cancer patients (Classified by glycemic Control) and nondiabetic patients.

Factor	FBG < 6.1 (*n* = 125)	FBG 6.1–9.9 (*n* = 289)	FBG ≥ 10.0 (*n* = 48)	No diabetes (*n* = 462)	p1^#^	p2^†^	p3^‡^
*ER*							
Positive	93 (74.4)	223 (77.2)	27 (56.2)	351 (76.0)	0.716	0.544	0.020^a^
Negative	32 (25.6)	66 (22.8)	21 (43.8)	111 (24.0)			
*PR*							
Positive	82 (65.6)	198 (68.5)	24 (50.0)	287 (62.1)	0.475	0.561	0.059
Negative	43 (34.4)	91 (31.5)	24 (50.0)	175 (37.9)			
*HER2*							
Positive	14 (11.2)	23 (8.0)	3 (6.2)	59 (12.8)	0.637	0.289	0.405
Negative	111 (88.8)	266 (92.0)	45 (93.8)	403 (87.3)			
*Ki-67*							
High	93 (74.4)	206 (71.3)	40 (83.3)	353 (76.4)	0.641	0.515	0.212
Low	32 (25.6)	83 (28.7)	8 (16.7)	109 (23.6)			
*Molecular subtype*							
Luminal A	25 (20.0)	60 (20.8)	4 (8.3)	84 (18.2)	0.643	0.860	0.066
Luminal B	67 (53.6)	161 (55.7)	23 (47.9)	271 (58.7)	0.310	0.692	0.503
HER2	9 (7.2)	16 (5.5)	3 (6.3)	37 (8.0)	0.765	0.514	1.000
TNBC	24 (19.2)	52 (18.0)	18 (37.5)	70 (15.2)	0.274	0.771	0.012^a^
*Histological grade*							
I	8 (6.4)	12 (4.2)	1 (2.1)	15 (3.2)	0.107	0.327	0.447
II	98 (78.4)	207 (71.6)	33 (68.8)	355 (76.8)	0.712	0.151	0.185
III	19 (15.2)	70 (24.2)	14 (29.2)	92 (19.9)	0.233	0.040^a^	0.036
*Lymph node metastasis*							
Positive	45 (36.0)	122 (42.2)	24 (50.0)	172 (37.2)	0.801	0.237	0.092
Negative	80 (64.0)	167 (57.8)	24 (50.0)	290 (62.8)			
*Pathological TNM staging*							
0 (DCIS)	9 (7.2)	13 (4.5)	1 (2.1)	16 (3.5)	0.066	0.261	0.287
I	37 (29.6)	72 (24.9)	7 (14.6)	165 (35.7)	0.202	0.320	0.042^a^
IIa	49 (39.2)	109 (37.7)	23 (47.9)	130 (28.1)	0.017^a^	0.775	0.298
IIb	12 (9.6)	46 (15.9)	6 (12.5)	54 (11.7)	0.512	0.089	0.584
IIIa	7 (5.6)	24 (8.3)	4 (8.3)	40 (8.7)	0.264	0.337	0.509
IIIb	0 (0)	1 (0.3)	0 (0)	0 (0)	NA	1.000	NA
IIIc	11 (8.8)	24 (8.3)	7 (14.6)	57 (12.3)	0.273	0.868	0.275

Data presented as n (%).

*FBG* fasting blood glucose, mmol/L.

^#^p1: comparison between diabetic with good glycemic control (FBG < 6.1 mmol/L) and nondiabetic patients.

^†^p2: comparison between diabetic with good glycemic control and diabetic with FBG 6.1–9.9 mmol/L.

^‡^p3: comparison between diabetic with good glycemic control and diabetic with FBG ≥ 10.0 mmol/L.

^a^Statistically significant difference.

### Diabetic treatment affects the molecular subtype of breast cancer

We also evaluated the influence of diabetes treatment on tumor characteristics. Among the 462 diabetic patients, those treated with metformin (*n* = 192) exhibited a higher incidence of TNBC compared to those not on metformin (26.6% vs. 15.9%; *p* = 0.005) and a lower ER positivity rate (69.3% vs. 77.8%; *p* = 0.039). No significant differences were noted in HER2 or Ki-67 expression, histological grade, TNM stage, or lymph node metastasis (Table 3).

**Table 3:** Histopathological characteristics of diabetic patients with and without Metformin and/or insulin Treatment.

Factor	Metformin Treatment Group (*n* = 192)	Control Group (*n* = 270)	*p*	Insulin Treatment Group (*n* = 160)	Control Group (*n* = 302)	*P*	Metformin + Insulin Treatment Group (*n* = 30)	Control Group (*n* = 140)	*p*
*ER*									
Positive	133 (69.3)	210 (77.8)	0.039^a^	124 (77.5)	219 (72.5)	0.244	20 (66.7)	106 (75.7)	0.305
Negative	59 (30.7)	60 (22.2)		36 (22.5)	83 (27.5)		10 (33.3)	34 (24.3)	
*PR*									
Positive	122 (63.5)	182 (67.4)	0.388	101 (63.1)	203 (67.2)	0.378	16 (53.3)	97 (69.3)	0.093
Negative	70 (36.5)	88 (32.6)		59 (36.9)	99 (32.8)		14 (46.7)	43 (30.7)	
*HER2*									
Positive	12 (6.3)	28 (10.4)	0.121	17 (10.6)	23 (7.6)	0.274	7 (23.3)	18 (12.9)	0.141
Negative	180 (93.7)	242 (89.6)		143 (89.4)	179 (92.4)		23 (76.7)	122 (87.1)	
*Ki-67*									
High	146 (76.0)	193 (71.5)	0.274	118 (73.8)	221 (73.2)	0.895	24 (80.0)	99 (70.7)	0.302
Low	46 (34.0)	77 (28.5)		42 (26.2)	81 (26.8)		6 (20.0)	41 (29.3)	
*Molecular subtype*									
Luminal A	38 (19.8)	51 (18.9)	0.808	27 (16.9)	62 (20.5)	0.343	3 (10.0)	27 (19.3)	0.266
Luminal B	94 (49.0)	157 (58.1)	0.051	95 (59.4)	156 (51.7)	0.113	17 (56.7)	79 (56.4)	0.981
HER2	9 (4.7)	19 (7.0)	0.297	7 (4.4)	21 (7.0)	0.269	5 (16.7)	17 (12.1)	0.503
TNBC	51 (26.6)	43 (15.9)	0.005^a^	31 (19.4)	63 (20.9)	0.706	5 (16.7)	17 (12.1)	0.502
*Histological grade*									
I	8 (4.2)	13 (4.8)	0.742	5 (3.1)	16 (5.3)	0.286	0 (0)	8 (4.9)	< 0.001^a^
II	141 (73.4)	197 (73.0)	0.910	122 (76.3)	216 (71.5)	0.275	29 (96.7)	94 (67.1)	0.001^a^
III	43 (22.4)	60 (22.2)	0.965	33 (20.6)	70 (23.3)	0.530	1 (3.3)	28 (20.0)	0.028^a^
*Lymph node metastasis*									
Positive	80 (41.7)	111 (41.1)	0.905	64 (40.0)	127 (42.1)	0.677	11 (36.7)	58 (41.4)	0.630
Negative	112 (58.3)	159 (58.9)		96 (60.0)	175 (57.9)		19 (63.3)	82 (58.6)	
*Pathological TNM staging*									
0 (DCIS)	14 (7.3)	9 (3.3)	0.054	16 (10.0)	7 (2.3)	< 0.001^a^	7 (23.3)	0 (0)	< 0.001^a^
I	53 (27.6)	63 (23.3)	0.297	24 (15.0)	92 (30.5)	< 0.001^a^	2 (6.7)	41 (29.3)	0.010^a^
IIa	73 (38.0)	108 (40.0)	0.668	67 (41.9)	114 (37.7)	0.387	11 (36.7)	52 (37.1)	0.961
IIb	18 (9.4)	46 (17.0)	0.019^a^	27 (16.9)	37 (12.3)	0.171	5 (16.7)	24 (17.1)	0.950
IIIa	20 (10.4)	15 (5.6)	0.052	11 (6.9)	24 (7.9)	0.679	3 (10.0)	7 (5.0)	0.384
IIIb	0 (0)	1 (0.4)	1.000	0 (0)	1 (0.3)	1.000	0 (0)	1 (0.7)	NA
IIIc	14 (7.3)	28 (10.4)	0.257	15 (9.4)	27 (8.9)	0.877	2 (6.7)	15 (10.7)	0.740

Data presented as n (%)

^a^Statistically significant difference.

In the subgroup treated with insulin (*n* = 160), patients showed a significantly lower incidence of pTNM stage I tumors (15.0% vs. 30.5%; *p* < 0.001) and a markedly higher rate of ductal carcinoma in situ (DCIS) (10.0% vs. 2.3%; *p* < 0.001). No significant differences in molecular subtype or lymph node involvement were detected when compared to non-insulin users. Patients receiving both metformin and insulin (*n* = 30) had a higher proportion of histological grade II tumors (96.7% vs. 67.1%; *p* = 0.001), a lower frequency of pTNM stage I disease (6.7% vs. 29.3%; *p* = 0.010), and a significantly higher rate of DCIS (23.3% vs. 0%; *p* < 0.001) compared to those receiving neither drug (Table 3).

### Influence of diabetes duration

To assess the impact of diabetes duration, patients were grouped into long-duration (**≥ 7** years; *n* = 209) and short-duration (< 7 years; *n* = 253**)** categories. The long-duration group had significantly lower frequencies of HER2-positive (2.9% vs. 8.7%; *p* = 0.009) and TNBC subtypes (15.8% vs. 24.1%; *p* = 0.027). They also had higher ER (82.8% vs. 67.2%; *p* < 0.001) and PR (71.3% vs. 61.3%; *p* = 0.024) positivity, a higher incidence of grade I tumors (6.7% vs. 2.8%; *p* = 0.043), and a lower rate of pTNM stage I tumors (20.1% vs. 29.2%; *p* = 0.024) (Table 4).

**Table 4:** Characteristics of patients classified by diabetes duration.

Factor	Disease duration ≥ 7 years (*n* = 209)	Disease duration < 7 years (*n* = 253)	*p*
*ER*			
Positive	173 (82.8)	170 (67.2)	< 0.001^a^
Negative	36 (17.2)	83 (32.8)	
*PR*			
Positive	149 (71.3)	155 (61.3)	0.024^a^
Negative	60 (28.7)	98 (38.7)	
*HER2*			
Positive	15 (7.2)	25 (9.9)	0.304
Negative	194 (92.8)	228 (90.1)	
*Ki-67*			
High	146 (69.9)	193 (76.3)	0.120
Low	63 (30.1)	60 (23.7)	
*Molecular subtype*			
Luminal A	48 (23.0)	41 (16.2)	0.067
Luminal B	122 (58.4)	129 (51.0)	0.113
HER2	6 (2.9)	22 (8.7)	0.009^a^
TNBC	33 (15.8)	61 (24.1)	0.027^a^
*Histological grade*			
I	14 (6.7)	7 (2.8)	0.043^a^
II	157 (75.1)	181 (71.5)	0.388
III	38 (18.2)	65 (25.7)	0.054
*Lymph node metastasis*			
Positive	92 (44.0)	99 (39.1)	0.288
Negative	117 (56.0)	154 (60.9)	
*Pathological TNM staging*			
0 (DCIS)	12 (5.7)	11 (4.3)	0.493
I	42 (20.1)	74 (29.2)	0.024^a^
IIa	88 (42.1)	93 (36.8)	0.241
IIb	34 (16.3)	30 (11.9)	0.172
IIIa	16 (7.7)	19 (7.5)	0.953
IIIb	0 (0)	1 (0.4)	1.000
IIIc	17 (8.1)	25 (9.9)	0.516

Data presented as n (%).

^a^Statistically significant difference.

### Multivariable analysis

To control for potential confounding variables, multivariable logistic regression was conducted adjusting for age, menopausal status, glycemic control, diabetes treatment, and diabetes duration. The analysis revealed that FBG ≥ 10 mmol/L and metformin treatment were independently associated with higher odds of TNBC (Table 5). In contrast, a diabetes duration ≥ 7 years was significantly associated with lower odds of HER2 positivity and TNBC. FBG ≥ 10 mmol/L was also independently associated with reduced ER and PR expression. Conversely, a longer diabetes duration (≥ 7 years) was associated with increased ER and PR positivity. No variables were found to be significantly associated with HER2 expression in the adjusted model (Table 5).

**Table 5:** The results of multinomial logistic regression Analysis.

Factor	Luminal B OR	*p*	95% CI	HER2 OR	*p*	95% CI	TNBC OR	*p*	95% CI
Age	1.010	0.472	0.983–1.038	0.998	0.941	0.950–1.048	0.997	0.879	0.965–1.031
FBG ≥ 10.0	2.352	0.158	0.718–7.705	1.418	0.698	0.243–8.270	5.704	0.007^a^	1.594–20.416
FBG 6.1–9.9	0.989	0.970	0.565–1.733	0.676	0.254	0.212–1.508	0.987	0.970	0.491–1.983
Metformin	0.906	0.717	0.531–1.546	0.502	0.164	0.190–1.324	1.966	0.043^a^	1.020–3.789
Insulin	1.481	0.171	0.844–2.598	0.758	0.602	0.268–2.147	1.918	0.068	0.952–3.865
Disease duration ≥ 7 years	0.761	0.279	0.464–1.248	0.221	0.003^a^	0.080–0.608	0.468	0.015^a^	0.253–0.864

Reference category: Luminal A subtype.

*CI*  confidence interval, *OR* odds ratio.

^a^Statistically significant difference.

## Discussion

This study retrospectively analyzed the clinico-pathological data of 924 breast cancer patients—462 diabetic and 462 non-diabetic—to characterize the association of T2DM with the molecular subtype and histopathological features of breast cancer in Chinese patients. The contributions of glycemic control, diabetes therapeutics, and the duration of diabetes to breast cancer were also assessed. Our results suggested a significantly higher incidence of TNBC and a lower incidence of HER2-positive breast cancer in the diabetic than the nondiabetic patients. This is partially consistent with previous findings in a cohort of 1021 patients, including 803 nondiabetic and 218 diabetic patients from Canada, which showed a lower HER2-positive rate in diabetic patients^16^. A study in Slovenian diabetic breast cancer patients also concluded that the occurrence of HER2-positive cancer was less frequent^19^. These findings collectively highlight the association of the diabetic metabolic condition with the molecular features of breast cancer.

At the molecular level, T2DM and breast cancer share common pathways involving insulin and insulin-like growth factor (IGF) signaling. An increase in insulin or IGF-1 levels, commonly seen in T2DM, is strongly associated with increased cancer risk and mortality^20–23^. IGF signaling is known to be activated in TNBC tumors and cell lines^24^. Both insulin and IGF-1 can promote cell proliferation and inhibit apoptosis in breast cancer cells, even at physiologically relevant concentrations^25,26^. In breast cancer tissues, the “A” isoform of the insulin receptor (IR-A) is upregulated and can be activated by insulin or IGF^27^. Moreover, the PI3K and/or Ras/MAPK pathways downstream of IR-A are known to promote tumor proliferation^28^. Although previous studies have suggested a possible link between IGF signaling and HER2 expression^29^, our data showed a lower HER2 rate in diabetics, indicating that additional mechanisms or tumor heterogeneity may be involved. This discrepancy highlights the need for further mechanistic investigations.

Our data further support a link between glycemic control in diabetic breast cancer patients and tumor molecular characteristics. Patients with poor glycemic control (FBG ≥ 10 mmol/L) exhibited a significantly higher risk of TNBC and histological grade III tumors, as well as lower ER expression and fewer stage I diagnoses. These findings suggest that dysregulated glucose metabolism may influence tumor aggressiveness in non-metastatic breast cancer.

The influence of diabetes treatment on breast cancer characteristics has also been extensively reported. Metformin, a common first-line therapy in T2DM, is recognized for its dual effects in lowering blood glucose and reducing hyperinsulinemia^30,31^. Studies suggest that metformin may improve neoadjuvant therapy responses^32^ and suppress breast cancer growth via insulin-lowering and AMPK activation^12,14^. However, in our study, diabetic patients treated with metformin had a significantly higher incidence of TNBC. This observation should be interpreted with caution, as we could not stratify metformin use based on timing, disease severity, or insulin resistance status. A similar TNBC association with metformin has been reported previously^19^. Patients with a diabetes history of ≥ 7 years prior to breast cancer diagnosis had a lower risk of TNBC and HER2-positive subtypes, but a higher frequency of ER- and PR-positive tumors and grade I tumors. This trend may reflect selection bias, altered tumor biology with long-term metabolic adaptation, or differences in treatment adherence. However, as this was a cross-sectional study, we could not determine the prognostic significance or survival outcomes associated with these differences.

To adjust for confounding factors such as age and menopausal status, we conducted multivariable logistic regression, which confirmed independent associations between FBG level, metformin use, diabetes duration, and specific breast cancer subtypes. However, other important confounding variables—such as body mass index (BMI), family history of breast cancer, physical activity, dietary habits, and metabolic syndrome—were not available in our dataset and thus could not be adjusted for, representing a key limitation of this study. Furthermore, as this was a cross-sectional analysis, no information on progression-free or overall survival was available, and causal inferences cannot be made. The findings represent associations observed at diagnosis rather than longitudinal trends. Therefore, prospective cohort studies with comprehensive clinical, lifestyle, and treatment data, along with long-term follow-up, are needed to validate these results and assess their prognostic implications. Additionally, as our study was conducted in a single tertiary cancer center in China, the generalizability of the findings to other populations may be limited.

## Conclusion

T2DM correlates with the risks of more aggressive subtypes such as TNBC and a lower incidence of HER2-positive breast cancer among Chinese patients. The status of glycemic control, treatment type, and duration of diabetes are significantly associated with the molecular subtype and histopathological characteristics of breast cancer. These findings suggest that metabolic factors may influence tumor biology, although causal relationships cannot be established due to the cross-sectional design. Further prospective studies with longitudinal follow-up are needed to assess the clinical outcomes and prognostic significance of these associations.

## Data Availability

Data is associated with this manuscript is available at figshare (https://figshare.com/) https://figshare.com/s/ca411e6b464309de25b2.
